# Identifying Systemically Important Companies by Using the Credit Network of an Entire Nation

**DOI:** 10.3390/e20100792

**Published:** 2018-10-16

**Authors:** Sebastian Poledna, Abraham Hinteregger, Stefan Thurner

**Affiliations:** 1 International Institute for Applied Systems Analysis, Schlossplatz 1, 2361 Laxenburg, Austria; 2 Complexity Science Hub Vienna, Josefstädter Straße 39, 1080 Vienna, Austria; 3 Section for Science of Complex Systems, Medical University of Vienna, Spitalgasse 23, 1090 Vienna, Austria; 4 Santa Fe Institute, 1399 Hyde Park Road, Santa Fe, NM 87501, USA

**Keywords:** credit network, systemic importance, bank-firm network, interbank network, systemic risk, financial regulation, contagion

## Abstract

The notions of systemic importance and systemic risk of financial institutions are closely related to the topology of financial liability networks. In this work, we reconstruct and analyze the financial liability network of an entire economy using data of 50,159 firms and banks. Our analysis contains 80.2% of the total liabilities of firms towards banks and all interbank liabilities in the Austrian banking system. The combination of firm-bank networks and interbank networks allows us to extend the concept of systemic risk to the real economy. In particular, the systemic importance of individual companies can be assessed, and for the first time, the financial ties between the financial and the real economy become explicitly visible. We find that firms contribute to systemic risk in similar ways as banks do. We identify a set of mid-sized companies that carry substantial systemic risk. Their default would affect up to 40% of the Austrian financial market. We find that all firms together create more systemic risk than the entire financial sector. In 2008, the total systemic risk of the Austrian interbank network amounted to only 29% of the total systemic risk of the entire financial network consisting of firms and banks. The work demonstrates that the notions of systemically important financial institutions (SIFIs) can be directly extended to firms.

## 1. Introduction

The financial crisis of 2007–2008 was triggered by the default of a single investment bank. The consequences of this default propagated through the financial system, bringing it to the brink of collapse. Because of close links between the financial and the real economy, the financial crisis spread quickly and was followed by a global economic downturn, the so-called Great Recession. The mechanisms of how a financial crisis may lead to an economic recession, and vice versa, are not understood on a fundamental level. To clarify and map the financial ties between the financial and the real economy, which are at the core of such potential spreading mechanisms, are more important than ever.

In response to the financial crisis, the Basel III framework recognizes systemically important financial institutions (SIFIs) and, in particular, global and domestic systemically important banks (G-SIBs or D-SIBs). For those, Basel III recommends increased capital requirements, so-called SIFI surcharges [1]. In this context, several network-based measures that identify systemically important financial institutions have been proposed and were recently applied [2,3,4,5,6,7,8]. These measures introduce the notion of the systemic importance of a financial institution within a financial network and are based on network centrality, or closely related measures. Network-based approaches typically work well for small financial networks (e.g., banking networks) with a relatively small number of financial institutions (nodes), usually less than a thousand. A serious disadvantage of many centrality measures is, however, that the values associated with particular institutions have no clear interpretation as a measure of expected losses. A solution that solves this problem is the so-called “DebtRank”, a recursive method suggested by Battiston et al. [2], that quantifies the systemic importance of financial institutions in terms of losses that the institution would contribute to the total loss in the system in the event of a default. Since data on financial networks are hard to obtain outside central banks, there have been several attempts to quantify the systemic importance of institutions without explicit knowledge of the underlying networks [9,10,11,12].

The vast majority of systemic risk analyses have focused on financial systems, with little emphasis placed on the real economy [13]. Driven by recent data availability, research on financial networks has focused on default contagion, mostly on direct lending networks between financial institutions [14,15,16,17,18,19,20,21,22] and, to a lesser degree, on derivative exposures [3,23]. Research on financial multi-layer networks that considers contagion channels in multiple financial asset markets (not only credit) emerged only recently. Poledna et al. [24] and León et al. [25] studied the interactions between financial institutions on different financial markets in Mexico and Colombia, respectively. Only a few works have studied the detailed relations between the financial and the real economy empirically. These focused on Japan and were mainly concerned with the topology of credit networks between banks and large firms [26,27,28,29]. De Masi et al. [30] and Miranda and Tabak [31] studied credit networks in Italy and Brazil, and Lux [32] developed a theoretical model of a bipartite credit network between banks and the non-bank corporate sector. De Masi et al. [30] and De Masi and Gallegati [27] used network analysis to study the credit networks in Italy and Japan, while  Fujiwara et al. [26] and Marotta et al. [29] investigated the evolution of the network structure in Japan. Marotta et al. [29] used community detection to identify communities of both banks and firms. Miranda and Tabak [31] and Aoyama [28] made a first attempt to analyze empirically systemic risk in credit networks in Japan and Brazil. Aoyama [28] used DebtRank to study risk propagation from banks to firms with a dataset, provided by Nikkei Inc. that contains approximately 2000 firms and 200 banks in Japan; however, it does not include interbank data. Miranda and Tabak [31] presented the first study that included interbank and firm loans. The used dataset was relatively small, as it contained only about 50 banks and 351 firms in Brazil.

In this work, we analyze a large financial credit network that not only includes all interbank liabilities, but also nearly all liabilities between banks and firms. We reconstruct the network by combing datasets that contain annual financial statements of nearly all firms and banks in Austria (approximately 170,000 firms and close to 1000 banks) with anonymized interbank liabilities from the Austrian banking system. This combined financial network of firms and banks allows us to identify systemically important firms by extending DebtRank to the combined financial networks. We estimate the share of systemic risk introduced by firms and compare the systemic risk levels of the interbank network with those of the combined financial network. It becomes obvious that the notion of SIFIs and G-SIBs, or D-SIBs, can be directly extended to firms.

The paper is structured as follows. Section 2 provides an overview of the datasets used in this study. In Section 3, we explain the methodology to reconstruct the entire financial network from data. In Section 4 and Section 5, we present the results by first presenting classical network statistics of the entire financial network, followed by an analysis of systemic importance of firms and banks. Finally, Section 6 discusses the results and provides conclusions.

## 2. Data

We use two data sources for the analysis: annual financial statements of nearly all firms and banks in Austria and anonymized interbank liabilities from the Austrian banking system. Financial statements of firms were obtained from the SABINAdatabase (The SABINA database is provided by Bureau van Dijk; see https://www.bvdinfo.com/en-us/our-products/company-information/national-products/sabina), which provides information on about 170,000 firms in Austria. This database contains detailed company financials for up to 10 years, as well as data on shareholders and subsidiaries, activity codes and trade descriptions and stock data for listed companies. The database includes bank-firm relations and allows us to identify which firm is a customer of which bank.

The financial statements of banks are made publicly available by the Austrian Central Bank (OeNB) (https://www.oenb.at/jahresabschlusski/jahresabschlusski). Interbank data provided by the OeNB contain fully-anonymized and linearly-transformed interbank liabilities from the entire Austrian banking system over 12 consecutive quarters from 2006–2008. The dataset additionally includes total assets, total liabilities, assets due from banks, liabilities due to banks and liquid assets (without interbank assets/liabilities) for all banks, again in anonymized form.

**Figure 1:**
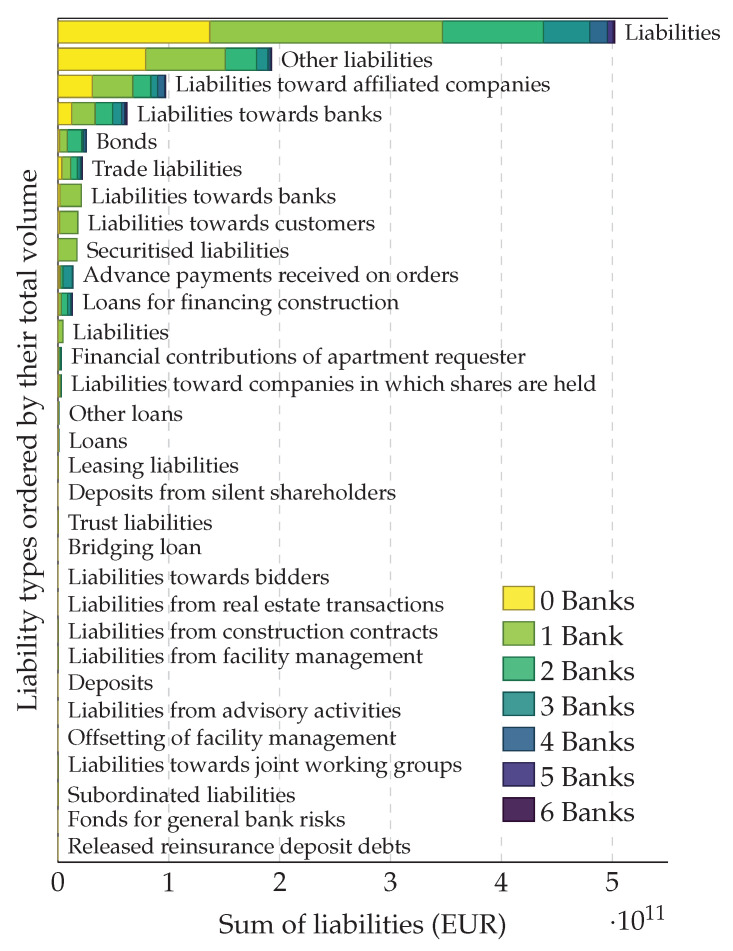
Aggregated debt structure of firms in Austria in 2008. The top bar shows the total liabilities ordered by the number of banks associated with each firm. The liabilities that are owed to a given number of banks (per firm) are shown in different colors. For instance, the liabilities of firms that receive loans from only one bank are shown in light green. The other stacked bars show the composition of different components of liabilities in the balance sheets. Different components of liabilities are sorted according to the total volume in each component.

A total of 106,919 firms and 796 banks filed a financial statement in the 2008 calendar year. Figure 1 shows the aggregated debt structure of firms in Austria in 2008. The top bar shows the total liabilities with respect to the number of lending banks associated with each firm. The other stack bars show the structure of different components of liabilities in the balance sheets.

**Figure 2:**
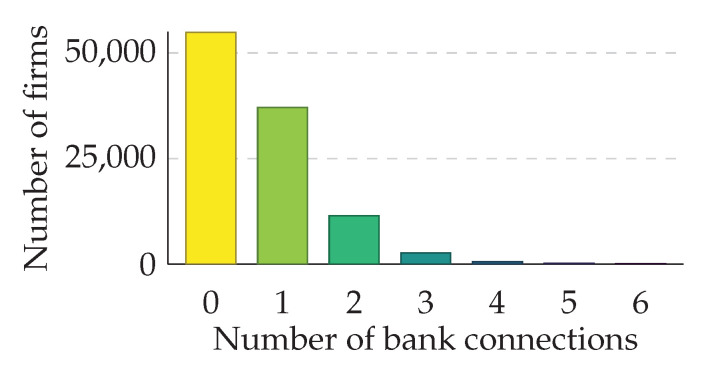
Number of banks associated with each firm. Different numbers of business relationships are shown in different colors. For instance, 37,079 firms have business relationships with only one bank.

In Figure 2, the number of banks associated with each firm is shown. Approximately 48.6% of the firms representing about 80.2% of total liabilities towards banks can be associated with one or more banks. Firms that cannot be associated with a bank are excluded from the analysis. For small firms that do not provide an exact breakdown of liabilities, we estimate liabilities towards banks by the average ratio of liabilities towards banks of firms in the same line of business, as indicated by their OeNACE code [33]. We reconstruct the liability network of 796 banks and 49,363 firms that contains 80.2% of the total liabilities of firms towards banks and all interbank liabilities.

## 3. Reconstruction of the Liability Network

**Figure 3:**
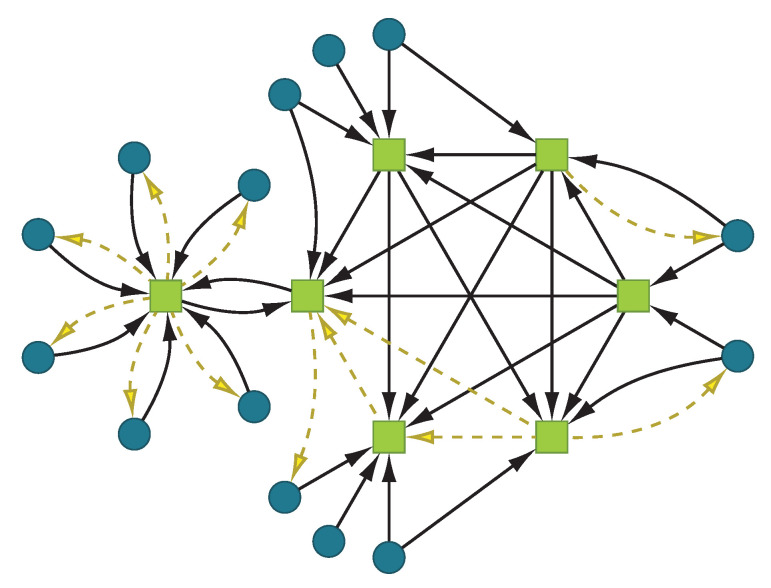
Schematic illustration of a network of banks 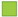 and firms 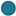. Connections are either loans or 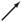 deposits 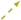. Banks are connected to firms and to each other, whereas firms only interact with banks.

We combined the two datasets to extract the bipartite network that represents the liabilities and assets of the Austrian economy. This network G=(F,E) consists of two disjunct sets of nodes: banks *B* and firms *C*, for which the trivial equations hold, B∪C=F, B∩C=∅,|B|=b and |C|=c. Links either connect banks with other banks (interbank liabilities), banks with firms (deposits of firms at banks) or firms with banks (liabilities of firms) as illustrated in Figure 3. The weighted adjacency matrix:

(1) $$\begin{array}{c} L_{n\times n}=\left(\begin{array}{cc} BB_{b\times b} & BC_{b\times c}\\ CB_{c\times b} & CC_{c\times c}=0 \end{array}\right),\;n=c+b\;, \end{array}$$

is called the liability matrix, where each entry $L_{ij}\in E$ indicates the liability that node *i* (which is a bank if i≤b and a firm if i>b) has towards node *j*. The matrix is partitioned into four parts:

1. Interbank network BB, connecting banks with banks.
2. Bank-firm network BC, containing information about deposits firms have at financial institutions.
3. Firm-bank network CB, containing information about liabilities firms have towards financial institutions (bank loans).
4. Firm-firm network CC with inter-firm liabilities, which are omitted in this work; thus, $CC_{xy}=0$ for all x,y∈[1,c].

The interbank network BB is obtained from the interbank dataset. In a first step, data on bank-firm relations are used to establish an unweighted bipartite network between firms and banks. This bipartite network is used as a basis for the BC and CB adjacency matrices, which are (after assigning weights; see below) combined with BB to obtain the liability network *L*. To match the interbank network BB with the bipartite bank-firm networks BC and CB, the banks of both datasets were ranked according to total assets. The resulting tables were then joined with their rank as a common column. In a second step, the weights of the bipartite liability network of firms and banks are assigned as follows:

- For every firm *c*, take the aggregated liabilities $L_{c}$ the firm has toward banks from the balance sheet.
- Then, take the set of aggregated loans (referred to as assets, or $A_{i}$, where *i* is the index of a bank/firm) of all banks from their balance sheets, and assign them to the entries of the vector *ℓ* in the following way:
  $$\ell_{b}=\left\{\begin{array}{cc} 0 & \mathrm{if}\;L_{cb}=0\\ A_{b} & \mathrm{else}. \end{array}\right.$$
- Normalize the resulting vector,
  $$\hat{\ell }=\frac{\ell }{\sum_{i}\left|\ell_{i}\right|}.$$
- Partition the aggregated liabilities with the distribution $\hat{\ell }$ to obtain the entries for the firm-bank network, $L_{:c}=L_{c}\cdot \hat{\ell }\;$, where we use vector notation and : means column.

Note that we partition the liabilities of each firm to their banks according to the relative size of the lending banks.

## 4. The Liability Network of Austria

**Figure 4:**
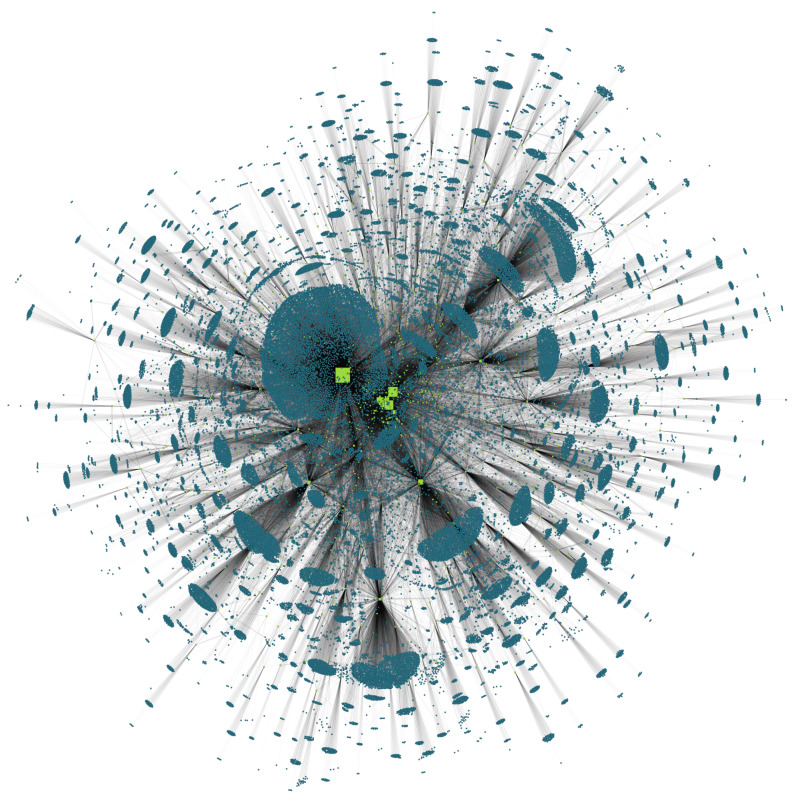
Reconstructed liability network of Austria with 796 bank nodes 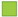 and 49,363 firm nodes 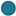 in 2008. The network represents approximately 80.2% of total liabilities towards banks of firms and all interbank liabilities. The node size corresponds to the total assets held by each node.

We use empirical data (see Section 2) to reconstruct the liability network of Austria, as outlined in Section 3. The resulting network with 50,159 nodes is visualized in Figure 4 and represents approximately 80.2% of total liabilities towards banks of firms and all interbank liabilities (we use the Hu Yifan network layout algorithm [34] in Gephi [35] for visualization). Bank nodes are represented by squares and firms by circles. The node size corresponds to the total assets held by each node.

Table 1 shows the directed and unweighted global clustering coefficients $\langle C_{i}\rangle$ of the entire liability network, as well as the interbank network. Clustering coefficients are significantly larger than those of the corresponding random graphs with an identical number of nodes and links.

**Figure 5:**
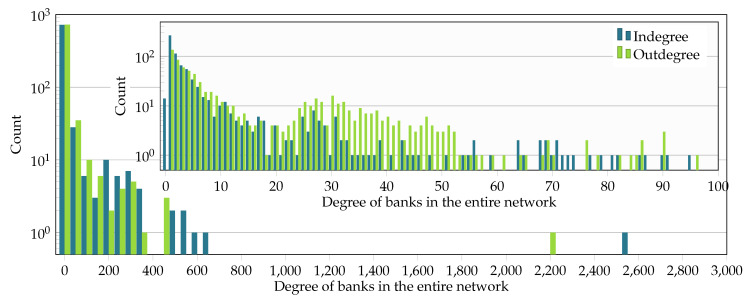
In- and out-degree of banks in the subgraph of 796 banks and 5000 firms. The main plot has 60 uniform bins on the interval [0,3000], while the inset shows the distribution of degrees in the range [0,100].

**Figure 6:**
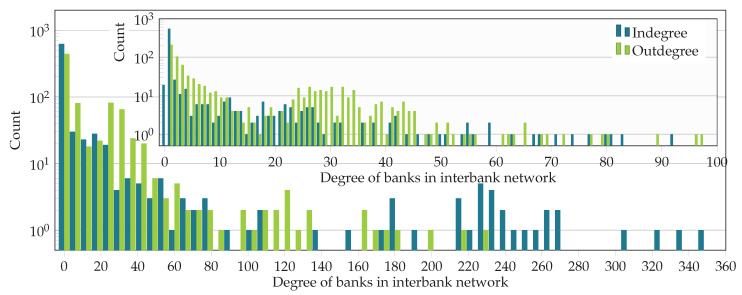
In- and out-degree of banks in the interbank network *B*. The main plot has 60 uniform bins on the interval [0,360], while the inset shows the distribution of degrees in the range [0,100].

For the following analysis, we chose the subgraph induced by the set of all 796 banks in the Austrian banking system and the 5000 firms with the highest liabilities. The degree distributions of the banks in the entire liability network and the interbank network are illustrated in Figure 5 and Figure 6. The in- and out-degree distributions are depicted in Figure 5 for the entire liability network *F* and in Figure 6 for the interbank network *B* only. In Figure 5 and Figure 6, the main plots show the whole degree range, and the insets provide a finer resolution in the ranges with higher density. Out-degrees are smaller than in-degrees, suggesting that highly interconnected banks provide interbank loans to more banks than from which they receive loans.

**Figure 7:**
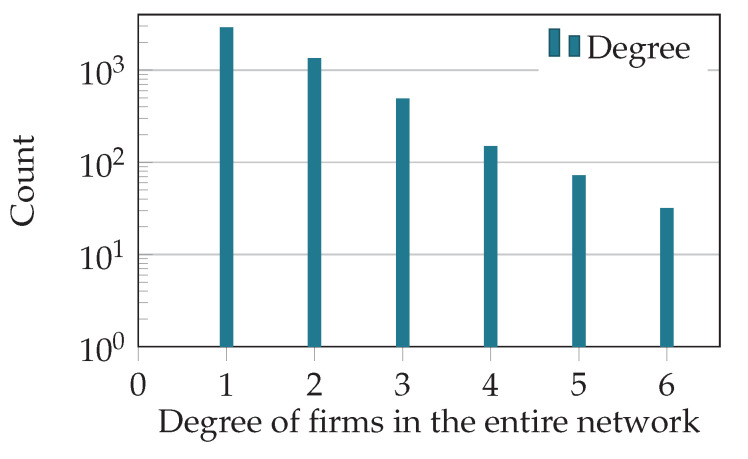
Degree of firms in the subgraph of 796 banks and 5000 firms. Values for in- and out-degrees are identical because loans and deposits (BC and CB network; see Section 3) use the same adjacency matrix that specifies the bank-firm connections in the commercial register.

Figure 7 shows the degree distribution of firms in the entire liability network (similar to Figure 2). The degree distribution is restricted to firms with degree >0 and contains the 5000 firms with the highest liabilities in 2008. Note that the in- and out-degree of firms are identical, since the bank-firm connections provided by the commercial register were used for deposits and liabilities.

## 5. Systemically Important Firms and Banks in Austria

**Figure 8:**
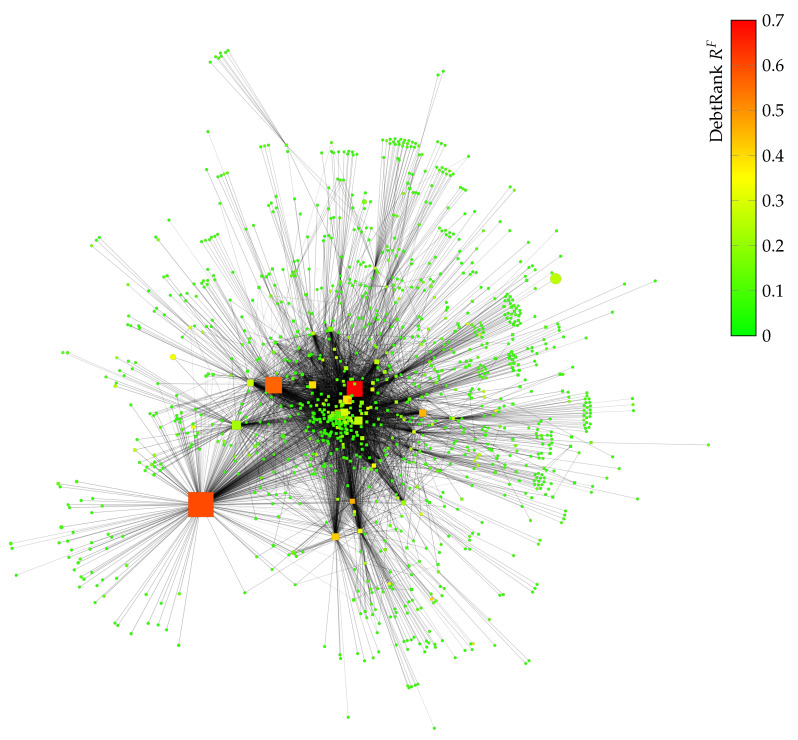
Subgraph of Figure 4 with nodes with a DebtRank $R^{F}\;\geq \;0.01$. Bank nodes are represented by squares and firms by circles. The node size corresponds to the total assets held by each node. Nodes are colored according to their DebtRank. Nodes representing firms and banks with more assets tend to have a higher DebtRank. However, there are also medium-sized banks and firms that show a high DebtRank.

To identify systemically important firms and banks, we use DebtRank. DebtRank is a recursive method to determine the systemic importance of nodes within financial networks [2]. It is a quantity, $R_{i}$ (or $R_{S}$), that measures the fraction of the total economic value *V*, in the network that is potentially affected by the distress of an individual node *i* (or by a set of nodes *S*). For details, see Appendix A. Figure 8 shows all banks (squares) and firms (circles) with a DebtRank $R^{F}\geq 0.01$. Node size represents the total assets, while the color encodes the DebtRank. Nodes with the highest DebtRank typically are large banks with substantial total assets. However, there are also several mid-sized banks and firms with a high DebtRank. Note that some mid-sized banks and firms (total assets below one billion EUR) have a very high DebtRank (≈0.4).

**Figure 9:**
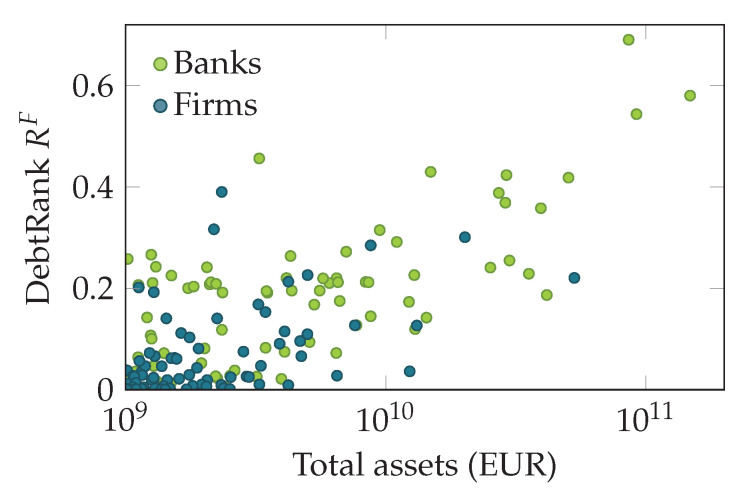
DebtRank of firms (dark green) and banks (light green) plotted against their total assets (as a proxy for firm size) in Euros. Note that firms with a similar DebtRank show differences in their asset sizes of multiple orders of magnitude. The distributions of banks and firms in the asset-DebtRank plain do not appear to be qualitatively different.

This can also be seen in Figure 9, which shows the DebtRank of firms and banks plotted in relation to their total assets. In general, firms, as well as banks with larger assets tend to have a higher DebtRank. However, firms with a similar DebtRank show a large variation in their total assets (multiple orders of magnitude). The distributions of banks and firms across the asset-DebtRank plain do not seem to be qualitatively different.

**Figure 10:**
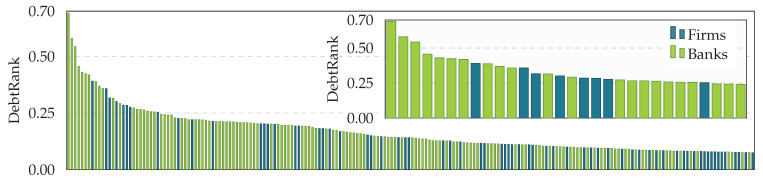
DebtRanks of 200 firms (dark green) and banks (light green) sorted by their DebtRank in decreasing order. The inset shows the 20 firms and banks with the highest DebtRank. The most systemically important nodes are banks; however, note that already the 8th most important node is a firm.

**Figure 11:**
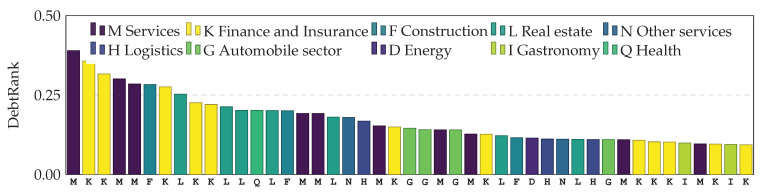
Firms in different economic sectors ranked by DebtRank (in descending order). Colors denote the economic sector of the firms. We follow the OeNACE classification (used as the x-axis label).

In Figure 10, we see 200 firms (dark green) and banks (light green) in Austria, ranked according to their systemic importance measured by DebtRank. It is not surprising that the most systemically important nodes are banks. It is, however, very interesting to find that the 8th most systemically important node already is a company. The DebtRank of that company is 0.39, meaning that the default of this firm would affect up to 39% of the Austrian financial system. Figure 11 shows the 45 firms with the highest DebtRank, where colors indicate their line of business according to the first level of their OeNACE code (below the bars) that is used to classify economic activities in Austria [33]. Systemically important firms are found across various industry sectors.

**Figure 12:**
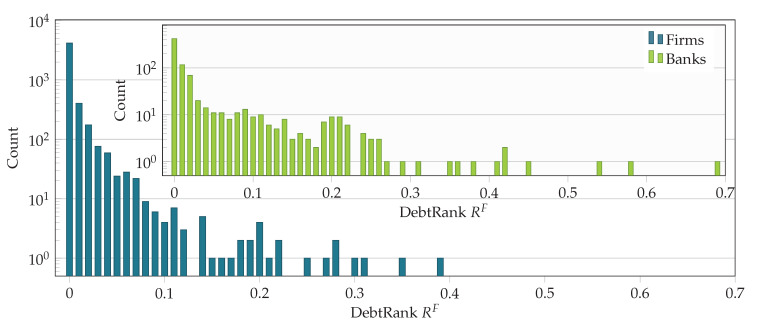
Histogram of DebtRank $R^{F}$ in the entire liability network of banks [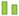] and firms [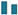]. Banks and firms have a qualitatively similar DebtRank distribution. The highest DebtRank of a firm is 0.39.

In Figure 12, the distribution of the DebtRank values of firms and banks (inset) is shown. Banks and firms have a qualitatively similar DebtRank distribution. Systemically important firms contribute systemic risk in a similar way as banks do.

Finally, we estimate the fraction of systemic risk contributed by firms in the entire liability network. We define $Q_{1}$ as the ratio of the sum of the DebtRank values of all firms, divided by the sum of all DebtRank values in the entire liability network (including banks),

(2) $$Q_{1}=\frac{\sum_{i\in C}R_{i}^{F}}{\sum_{i\in F}R_{i}^{F}}\;.$$

We find that $Q_{1}=0.55$ in Austria for 2008. Firms introduce more than half of the systemic risk in the entire liability network (more than banks). To compare the systemic risk of the interbank network with the systemic risk of the entire liability network, we define a similar ratio,

(3) $$Q_{2}=\frac{V^{B}\sum_{i\in B}R_{i}^{B}}{V^{F}\sum_{i\in F}R_{i}^{F}}\;,$$

where $V^{B}$ and $V^{F}$ refer to the total economic values of the interbank network and the entire liability network, respectively. In this case, we must take the different economic values of the two networks into account, since the DebtRank is a relative measure. We find $Q_{2}=0.29$ in Austria for 2008, that is the total systemic risk of the interbank network amounts to only 29% of the total systemic risk of the entire liability network.

## 6. Conclusions

The systemic importance of financial institutions is closely related to the topology of financial liability networks. In this work, we reconstruct and analyze the financial credit network of 50,159 firms and banks that contains 80.2% of the total liabilities of firms towards banks and all interbank liabilities in the entire Austrian banking system. The network allows us to understand the detailed credit linkage between the complete financial economy with a significant fraction of the real economy of an entire nation. To our knowledge, this is the most comprehensive financial network ever analyzed.

We find that firms introduce systemic risk in similar ways as banks. Banks and firms qualitatively show similar distributions of systemic importance. In particular, we identify several mid-sized banks and firms (with total assets below one billion EUR) in Austria that are systemically important in the entire financial network. The systemic importance of these firms is primarily driven by their position in the network. Moreover, systemically important firms are not associated with specific industrial sectors, but are spread across many different industries. We find that banks and firms of similar systemic importance (DebtRank) show a large variance in asset sizes that spans several orders of magnitude. Our main result is that firms introduce more systemic risk than the financial sector. The total systemic risk of the Austrian interbank network in 2008 amounted to only 29% of the total systemic risk of the entire financial network consisting of firms and banks.

These results come with three caveats due to partially missing and partly inaccurate data. First, the analyzed financial network had to be reconstructed from balance sheet data and could not be directly assessed from empirical data sources. The uncertainty in the reconstruction arises in the estimation of the weights (size of firm liabilities towards banks) of the (unweighted) adjacency matrix. The latter is directly observable in the empirical data. For a large subset of firms, the liabilities (42.4% of total liabilities towards banks) are known exactly and do not need to be reconstructed, since these firms are only customers of one bank (Figure 2). In addition, the interbank liabilities did not have to be reconstructed. Moreover, systemic risk, as measured by DebtRank, does seem to be rather robust against variations of reconstructed networks [36]. Second, interbank liabilities from the Austrian banking system are fully anonymized and linearly transformed. Thus, there remains a small uncertainty in the absolute value of the interbank liabilities, which also introduces some uncertainty in the matching process of the various datasets. Third, our analysis involves only one snapshot of the Austrian financial system in 2008, which is the only year where the two datasets overlap in time.

It would be interesting to extend this study to other countries and to investigate the evolution of similarly large financial networks that cover the financial and the real economy of an entire nation. It is a first step towards understanding the detailed linkage between the financial industry and the real economy. We believe that without that knowledge, it will remain hard to estimate the influence of financial crises on the real economy; in particular, under which circumstances a financial crisis will lead to an economic downturn and when will it not. The opposite question might also become answerable: Given an economic crisis, under which circumstances will it cause financial distress that might become systemic? Further investigation is needed to confirm and deepen the findings with other countries and across longer time horizons. However, we believe that it is clear from this contribution that the notion of systemically important financial institutions (SIFIs) or global and domestic systemically important banks can be directly extended to companies in the real economy. In Austria, we identify several mid-sized firms that carry substantial systemic risk, a fact that was hitherto not known. In conclusion, our analysis suggests that not only systemically important financial institutions, but also systemically important firms should be subject to macro-prudential regulation. 

## Figures and Tables

**Table 1:** Number of nodes and links in the entire liability network and in the interbank network alone. The directed and unweighted global clustering coefficient $\langle C_{i}\rangle$ for both networks show much higher clustering than the corresponding random graphs (same number of nodes and links).

Network	Nodes	Links	$\langle C_{i}\rangle$	${\langle C_{i}\rangle }^{\mathrm{rand}}$
Entire network *F*	50,159	140,528	0.126	0.001
Interbank network *B*	796	12,783	0.337	0.005

## Appendix A. DebtRank

The financial dependencies of the nodes in the network are given in a liability matrix *L* with entries, $L_{ij}$, denoting that node *j* has given node *i* a loan (or investment/deposit) of size $L_{ij}$. A capital (or equity) vector *C* with entries $C_{i}$ contains the capital of node *i*. The relative economic value of a node *i* is given by:

(A1) $$v_{i}=\frac{L_{i}}{\sum_{j}L_{j}},$$

where $L_{i}=\sum_{j}L_{ji}$ is the sum of the outstanding liabilities of node *i*. The default of node *i* then affects all nodes *j*, where $L_{ij}>0$. The impact of the default of *i* on *j* is defined as:

(A2) $$W_{ij}=\min\left(\frac{L_{ij}}{C_{j}},1\right)\;.$$

The impact of a shock is thus measured as the fraction of capital loss due to the credit default. It is therefore a value in the range [0,1]. $W_{ij}=0$ means that the default of node *i* does not affect node *j*, while $W_{ij}=1$ means that the default of node *i* results in a loss that matches or exceeds the capital of node *j*.

The economic value of the impact is obtained by multiplying the impact with the relative economic value from Equation (A1). The economic value of the impact of *i* on its neighbors is therefore given by:

(A3) $$I_{i}=\sum_{j}W_{ij}v_{j}\;.$$

If the neighbors of *i* do not have enough capital to compensate for the default of *i*, they default themselves and might cause an impact on their neighbors, as well as reverberations in the network, along paths in the impact network *W*. To prevent cycles (no bank defaults more than once), Battiston et al. [2] proposed to consider paths without repeating links. This is achieved by introducing two time-dependent state variables for each node, $s_{i}\left(t\right)$ and $h_{i}$(t). $s_{i}$ takes one of three values:

$s_{i}\left(t\right)$	**Interpretation**
U	Node *i* is undistressed at time *t*
D	Node *i* is in distress at time *t*
I	Node *i* is inactive at time *t*

The variable $h_{i}$ has a value within the range [0,1] and is known as the level of distress. $h_{i}=0$ means undistressed, and $h_{i}\left(t\right)=1$ signals the case of default. The value of $h_{i}\left(t\right)$ is defined as:

(A4) $$\begin{array}{cc} h_{i}\left(t\right) & =\min\left(1,h_{i}\left(t-1\right)+\sum_{j|s_{j}\left(t-1\right)=D}W_{ji}h_{j}\left(t_{1}\right)\right)\;, \end{array}$$

whereas $s_{i}\left(t\right)$ is given by:

(A5) $$\begin{array}{cc} 1-1s_{i}\left(t\right) & =\left\{\begin{array}{cc} D & \mathrm{if}\;h_{i}\left(t\right)>0\wedge s_{i}\left(t-1\right)\neq I,\\ I & \mathrm{if}\;s_{i}\left(t-1\right)=D,\\ s_{i}\left(t-1\right) & \mathrm{otherwise} \end{array}\right. \end{array}$$

To calculate the DebtRank of a node *d* (*d* for defaulting), the distress $h_{i}$ and status $s_{i}$ at time step t=1 are initialized as follows:

(A6) $$\begin{array}{cc} h_{i}\left(1\right) & =\left\{\begin{array}{cc} 1 & \mathrm{if}\;i=d\\ 0 & \mathrm{otherwise} \end{array}\right. \end{array}$$

(A7) $$\begin{array}{cc} s_{i}\left(1\right) & =\left\{\begin{array}{cc} D & \mathrm{if}\;i=d\\ U & \mathrm{otherwise}. \end{array}\right.\;. \end{array}$$

Then, the values of $s_{i}$ and $h_{i}$ are calculated for every node *i* and time step *t*, according to Equations (A4) and (A5), until all nodes are either inactive or undistressed at t=T. The DebtRank of node *d* can then be calculated as the sum of the distress in the whole network at time t=T, reduced by the distress at the beginning, that is the initial distress of node *d* at t=1:

(A8) $$R_{d}=\sum_{i}h_{i}\left(T\right)v_{i}-h_{d}\left(1\right)v_{d}.\;$$

It is possible to calculate the DebtRank of a set *S* of simultaneously defaulting nodes by replacing the i=d conditions in the initialization Equation (A7) by i∈S, and changing Equation (A8) to one of the following equations:

(A9) $$\begin{array}{cc} R_{S} & =\sum_{i}h_{i}\left(T\right)v_{i}-\sum_{d\in S}h_{d}\left(1\right)v_{d} \end{array}$$

(A10) $$\begin{array}{cc} R_{S} & =\sum_{i}h_{i}\left(T\right)v_{i}. \end{array}$$

Equation (A9) excludes the impact of the initial shock, whereas Equation (A10) does not. In this work, DebtRank is calculated for two different networks, the interbank network *B* and the entire liability network *F*. We use $R^{F}$ and $R^{B}$ to discriminate between the two.
